# Influence of Malondialdehyde and Matrix Metalloproteinase-9 on Progression of Carotid Atherosclerosis in Chronic Renal Disease with Cardiometabolic Syndrome

**DOI:** 10.1155/2015/614357

**Published:** 2015-10-11

**Authors:** Senija Rašić, Damir Rebić, Sabaheta Hasić, Ismar Rašić, Marina Delić Šarac

**Affiliations:** ^1^Clinic for Nephrology, Clinical Center University Sarajevo, Bosnia and Herzegovina; ^2^Department of Medical Biochemistry, Faculty of Medicine University of Sarajevo, Bosnia and Herzegovina; ^3^Clinic for Abdominal Surgery, Clinical Center University Sarajevo, Bosnia and Herzegovina; ^4^Clinic for Immunology University Clinical Centre Sarajevo, Bosnia and Herzegovina

## Abstract

Objective was to assess whether the concentration of malondialdehyde (MDA) as a marker of lipid peroxidation and serum concentration of matrix metalloproteinase-9 (MMP-9) are involved in the process of atherosclerosis in chronic kidney disease (CKD) patients nondialysis-dependent and those on peritoneal dialysis (PD), both with signs of cardiometabolic syndrome (CMS). Thirty CKD and 22 PD patients were included in a study. All observed patients were divided into three subgroups depending on the degree of atherosclerotic changes in the carotid arteries (CA). Severity of atherosclerotic changes in the CA was evaluated by ultrasonography. We confirmed significantly lower level of serum MDA throughout all the stages of atherosclerosis in PD patients compared with observed CKD patients (*P* < 0.05) and increased serum concentration of MDA and MMP-9 with the progression of severity atherosclerotic changes in both groups of patients. The multiple regression analysis revealed that MDA and MMP-9 are significant predictors of changes in IMT-CA CKD patients (*P* < 0.05) and plaque score on CA in these patients (*P* < 0.05). The results suggest that MDA and MMP-9 could be mediators of CKD-related vascular remodeling in CMS.

## 1. Introduction

Cardiovascular disease is one of the major causes of morbidity and mortality worldwide, especially among patients of chronic kidney disease (CKD) [1, 2]. There is an increasing evidence of cardiometabolic syndrome (CMS) involvement in CKD [3, 4], but a causal relationship has not been proven. Cardiometabolic syndrome is a cluster of metabolic abnormalities combining obesity with 2-3 risk factors which include insulin resistance, hypertension, and high triglyceride or low high density lipoprotein (HDL) serum levels. It also increases the risk of cardiovascular disease (CVD) and type 2 diabetes [5]. 

It is believed that atherosclerotic changes in blood vessels in chronic renal diseases contribute significantly to the high cardiovascular morbidity and mortality. Morphological and functional abnormalities of the endothelium are considered as prodromal stage of atherosclerosis and early marker of CVD [6] that facilitate the progress of atherosclerosis [7, 8] and contribute to the development of hypertension through the enhancement of vascular resistance. On the other hand, arterial calcifications are a significant risk factor for cardiovascular mortality in the general population. There is a strong evidence supporting the view that atherosclerosis is a disease characterized by low-level vascular inflammation [9–11]. Inflammation, inflammatory action of local stimuli such as products of the oxidation process and glycation end products, oxidative stress, and degradation of extracellular matrix (ECM) change vasculature in terms of development of atherosclerosis. 

Renal disease is associated with a graded increase in oxidative stress (OS) markers even in early CKD [12]. Oxidative stress can accelerate renal injury progression and contribute increasing cardiovascular risk. Some studies have documented that peritoneal dialysis is associated with decreased levels of oxidative stress and inflammatory markers compared to haemodialysis [13]. A small number of trials have been carried in order to determine associations between oxidative stress with vascular structure and function with equivocal results [14, 15]. On the other hand, uncontrolled expression of matrix metalloproteinases (MMPs), enzymes that degrade extracellular matrix (ECM), can result in tissue damage and the development of a number of destructive diseases such as arthritis, atherosclerotic plaque rupture, aortic aneurysm, and progression of tumors [16, 17].

However, the relevance of malondialdehyde as a marker of oxidative stress which is generated by peroxidation of unsaturated fats as well as the role of matrix metalloproteinase-9 in atherosclerosis progression in patients (pts) with chronic kidney disease (CKD) not yet on dialysis compared to patients on peritoneal dialysis is less known, particularly with respect to cardiometabolic syndrome.

The aim of this paper was to examine whether the serum concentration of malondialdehyde and matrix metalloproteinase-9 is involved in the process of atherosclerosis in patients with CKD not yet dialysis-dependent and those on peritoneal dialysis (PD) with signs of CMS.

## 2. Patients, Materials, and Methods

### 2.1. Study Population

This cross-section study was conducted at the Clinic for Nephrology, Clinical Center University in Sarajevo, from June 2014 through December 2014. Fifty-two adult patients with CMS and chronic kidney disease were included in the study. The subjects were divided into CKD patients not yet dialysis-dependent (30 pts; eGFR <60> 15 mL/min/1.73 m^2^) and patients on peritoneal dialysis for >6 months (22 pts). Antioxidants had not been taken by any subjects in the two groups. All PD patients underwent 4 to 5 dialysis changes with 2 liters of dialysis solution. The control group consisted of 20 age- and sex-matched healthy subjects.

Subjects who had an episode of peritonitis within the previous 3 months and patients with evidence of malignancy, autoimmune disease or chronic liver disease, active infection, history of cardiovascular or peripheral vascular disease, and diabetes and recent treatment with iron were excluded from the study.

Depending on the degree of severity of atherosclerotic changes in the carotid arteries all observed group patients were divided into three subgroups (without atherosclerosis (AS0), moderate atherosclerosis (AS1/2), and severe atherosclerosis (AS3)).

Kidney function was assessed by using estimated glomerular filtration rate (eGFR). Estimated glomerular filtration rate was performed using the MDRD (abbreviated Modification of Diet in Renal Disease equation GFR) [18]. Body mass index (BMI) was calculated as the ratio of body weight in kilograms and the square of height in meters (BMI = kg/m^2^). Body weight for BMI in PD patients is measured with dry abdomen (without dialysate solution). The blood pressure (BP) was measured with mercury sphygmomanometer after 15 minutes of rest, according to recommendation by the British Hypertension Society [19]. Hypertension was defined as systolic BP ≥140 mmHg and diastolic BP ≥90 mmHg or use of antihypertensive medications. 

Informed consent was obtained from all participants and the local ethics committee approved the study.

### 2.2. Measurement of Serum Concentration of Malondialdehyde (MDA) and Matrix Metalloproteinase-9 (MMP-9) 

All serum samples were stored at −80°C until they were measured.

The concentration of malondialdehyde (MDA) was analyzed at the Center for Cytogenetics and Molecular Medicine at the Medical Faculty in Sarajevo using a competitive enzyme immunoassay test (ELISA), which was performed with commercial kit for the assessment of the overall level of MDA (manufacturer: USCN Life Science Inc., US-CEA597GE). Reading of the results is carried out at 450 nm on a plate reader STAT FAX 2100, USA. The measurement concentration of MAD was expressed in nanograms per milliliter (ng/mL).

The concentration of the enzyme matrix metalloproteinase-9 (MMP-9) in serum was quantified by ELISA at the Clinic for Immunology University Clinical Centre Sarajevo, according to manufacturer's instructions (R&D Systems, Inc., RD- DMP900). Reading of the results is carried out by spectrophotometry at 450 nm (reader BIOTEK ELX50), with the correction wavelength at 540 nm or 570 nm. The measurement concentration of MMP-9 was expressed in nanograms per milliliter (ng/mL).

### 2.3. Ultrasound Examination of the Carotid Arteries

The severity of carotid artery atherosclerosis was evaluated using the mean common carotid artery (CA), intima media thickness (IMT), and plaque score (PS). Carotid ultrasonography was used to evaluate the mean IMT and the PS. High-resolution B mode and color Doppler and pulse Doppler ultrasonography of both carotid arteries were performed with an ultrasound scanner (Wall-Track system: W-T, Maastricht, the Netherlands) equipped with a 7.5-MHZ linear array transducer. Measurement was done by the same angiologist who was not familiar with the clinical status of the study patients. Patients were examined in the supine position with the head tilted backwards. After the carotid arteries were located by transverse scans, the probe was rotated 90° to obtain and record a longitudinal image of the anterior and posterior walls. The high-resolution images of the walls of the bilateral CA, internal carotid arteries (ICA), and carotid bulbs were examined according to recommendations of the American Society of Echocardiography Carotid Intima Media Thickness Task Force [20]. The IMT was defined as the distance between the leading edge of the lumen-intima echo and the leading edge of the media-adventitia echo in plaque-free area. At least three measurements (A, B, and C) were taken over one centimeter length of each wall segment CA, and these measurements on both sides were collected and divided to obtain the mean IMT.

The PS was calculated by adding the maximal thickness in millimeters of plaques in each segment on both sides (A + B + C + thickness of the contralateral carotid artery plaques). The length of individual plaques was not considered in determining the PS.

The presence of atherosclerosis in CCA is estimated as recommended by the Mannheim consensus about atherosclerosis [21]:

(1) without atherosclerosis (AS0): IMT less than 80% of the reference interval (RI), age- and gender-adjusted, with RI values obtained from previously published studies of monitoring, using the same ultrasound procedures;
(2) mild atherosclerosis (AS1): IMT > 80% of the RI;
(3) moderate atherosclerosis (AS2): the presence of carotid plaque, with no significant stenosis (PSV <125 cm/s);
(4) severe atherosclerosis (AS3): the presence of carotid plaque with threatening stenosis (PSV >125 cm/s).

### 2.4. Statistical Analysis

All data were expressed as the mean ± SD or as median and interquartile range. The distribution of variables was tested by the Kolmogorov-Smirnov and/or Shapiro-Wilk test. Student's* t*-test was used to compare the means of variable with normal distribution. Kruskall-Wallis test was used for statistical evaluation of more than 3 groups. The difference in median with interquartile range between two groups was analyzed by the Mann-Whitney test. Pearson's test was used to correlate data with normal distribution and Spearman's test for data with a skewed distribution. A multiple regression analysis was applied to define the independent connection serum concentration of MDA and MMP-9 with ultrasound parameters of CA.

The significant independent variables were determined according to their standardized effect, defined as regression coefficient/standard error of the regression (*β*).* P* values of <0.05 were considered statistically significant. All statistical calculations were performed with the SPSS 16 software (version 16.0, SPSS Inc., Chicago, IL, USA).

## 3. Results

### 3.1. Demographic Data

The average age of respondents was 59.84 ± 5.16 years and was not different from the age of the control group. There was also no difference in age and smoking in CMS patients with CKD without the need for dialysis treatment and those on peritoneal dialysis.

All monitored CKD and PD patients were overweight, while BMI of control group patients was within the limits that are considered healthy (between 20.1 and 24.5). In our study, all observed CKD patients, dependent or not dependent on dialysis treatment, were hypertensive (BP ≥140/90 mm Hg), but significantly higher values of both systolic and diastolic blood pressure were registered in the group of patients on PD treatment compared to other CKD patients. Although there were no significant intergroup differences in serum triglyceride levels, all groups of patients had an elevated level of serum triglycerides (above the recommended value of 1.7 mmol/L).

Basal characteristics of the anthropometric profile, blood pressure values, metabolic pattern, and serum concentration of MDA and MMP-9 in the whole group of CKD and PD subjects with CMS and in two subgroups of CKD with and without dialysis (PD) are presented in Table 1.

### 3.2. Analysis of MDA and MMP-9 Serum Concentration in the Dialysis and Nondialysis Patient Subgroups According to the Stage of Atherosclerosis

Depending on the stages of atherosclerosis our data showed significant changes in serum concentration of MDA and MMP-9 between the different subgroups of CKD nondialysis-dependent patients, as well as in patients on peritoneal dialysis (Table 2).

### 3.3. Analysis of MDA and MMP-9 Serum Concentration between CKD and PD Patients According to the Stage of Atherosclerosis

**Figure 1:**
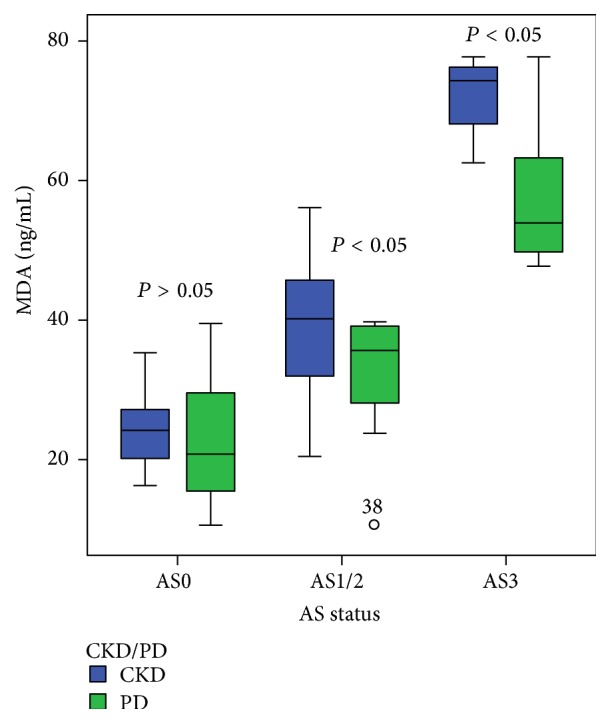
The relationship between serum concentrations of MDA in CKD nondialysis-dependent patients and PD patients according to the stage of atherosclerotic changes in the carotid arteries. Data are presented as median with interquartile range. Bars show the maximum and minimum value, while the square and its central bar show median and interquartile range. Note: AS: atherosclerosis.

The level of serum MDA through all the stages of atherosclerosis was significantly lower in PD patients compared to observed CKD nondialysis-dependent patients (in AS1/2 35.6 (28.3–39.1) versus 40.3 (31.9–45.6) ng/mL; *P* < 0.05 and in AS3 53.9 (49.8–63.2) versus 74.3 (68.2–76.3) ng/mL; *P* < 0.05). There were no significant differences in serum concentration of MDA in the monitored PD and CKD patients without expressed atherosclerotic changes (AS0 20.6 (10.4–31.3) versus 24.1 (18.8–27) ng/mL; *P* > 0.05) (Figure 1).

**Figure 2:**
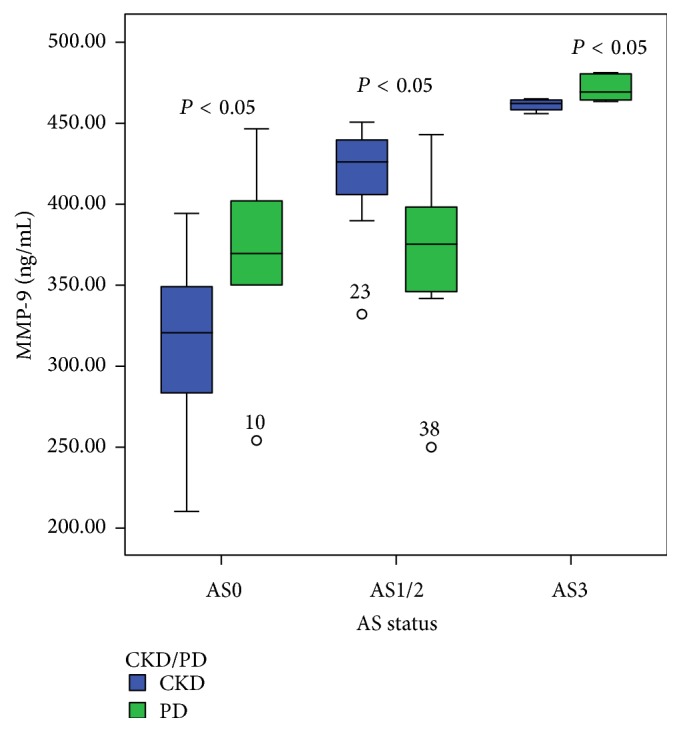
The relationship between serum concentrations of MMP-9 in CKD nondialysis-dependent patients and PD patients according to the stage of atherosclerotic changes in the carotid arteries. Data are presented as median with interquartile range. Bars show the maximum and minimum value, while the square and its central bar show median and interquartile range. Note: AS: atherosclerosis.

The level of serum concentration of MMP-9 was significantly different in PD patients compared to CKD nondialysis-dependent patients. Significantly lower level of this biomarker is registered in the stage AS1/2 of atherosclerosis in PD patients (374.8 (346.4–397.7) ng/mL) compared to the CKD patients treated conservatively (425.2 (405.5–439.5) ng/mL) (*P* < 0.05), whereas in stage AS3 serum MMP-9 concentration was significantly higher in PD patients in comparison with other CKD patients (*P* < 0.05). Such a relationship but lower concentration of MMP-9 in the serum was present in stage AS0 in both groups (Figure 2).

**Figure 3:**
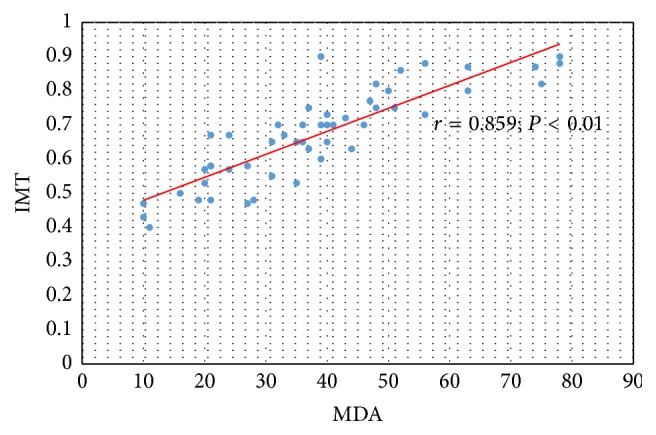
The relation between serum concentration of MDA and IMT-CA in CKD and PD patients.

**Figure 4:**
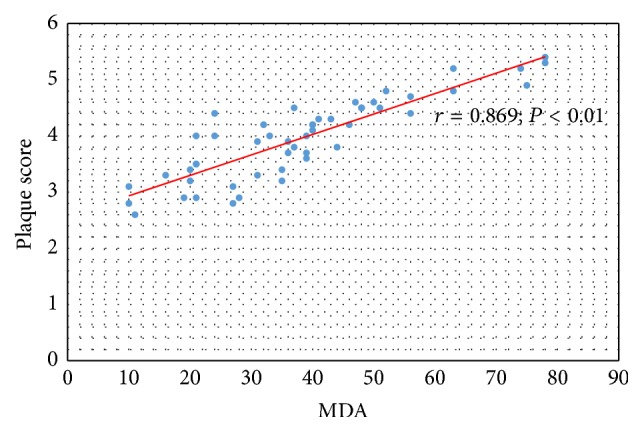
The relation between serum concentration of MDA and value of plaque score in CKD and PD patients.

Significant correlation was confirmed between serum concentration of MDA and IMT-CA in all observed patients (*r* = 0.859; *P* < 0.01) (Figure 3), as well as between serum concentration of MDA and value of plaque score (rho = 0.869; *P* < 0.01) (Figure 4).

**Figure 5:**
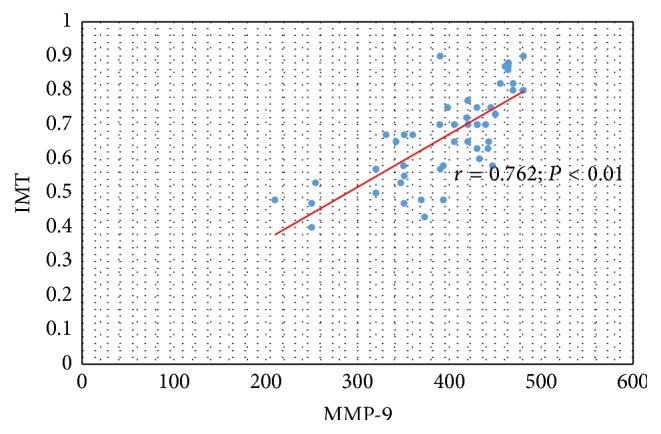
The relation between serum concentration of MMP-9 and IMT-CA in CKD and PD patients.

**Figure 6:**
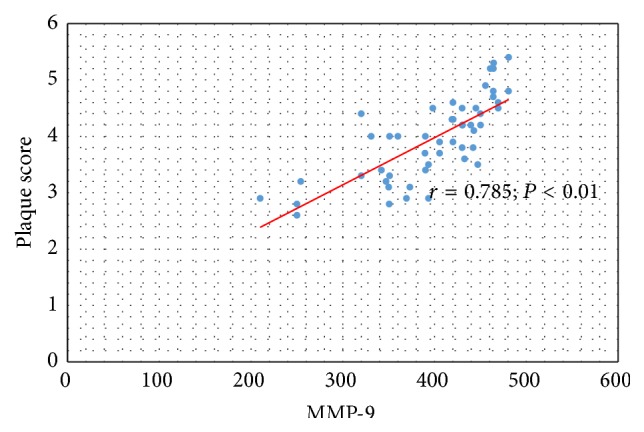
The relation between serum concentration of MMP-9 and value of plaque score in CKD and PD patients.

Significant relation was also confirmed between serum concentration of MMP-9 and IMT-CA (*r* = 0.762; *P* < 0.01) (Figure 5) and between serum concentration of MMP-9 and value of plaque score in all observed CKD and PD patients (*r* = 0.785; *P* < 0.01) (Figure 6).

In multiple regression model serum concentrations of MDA and MMP-9 were significant predictors of IMT-CA in all monitored CKD and PD patients (*P* < 0.05), as well as plaque score on carotid arteries in these populations (*P* < 0.05). This model is able to explain 70% of the variance in the results of IMT (*R* ^2^ = 0.765) and about 79% (*R* ^2^ = 0.786) of the variation that occurs with the plaque score on carotid arteries in chronic kidney disease (Table 3).

## 4. Discussion

Chronic kidney disease is regarded as a prooxidant and low-grade inflammation state. The degree of intracellular and extracellular oxidative stress is related to the severity of renal failure [22]. It was also noted that tissue damage caused by lipid peroxidation plays an important role in the developmentof various diseases including atherosclerosis, which is associated with high cardiovascular morbidity and mortality in CKD [23, 24]. In recent years a few studies were published dealing with the status of oxidative stress in relation to various disorders that accompany chronic renal disease and renal replacement therapy [25–29].

In the present paper we demonstrated that the serum concentration of MDA did not statistically differ in CKD patients with CMS treated conservatively and patients undergoing peritoneal dialysis. However, it was observed that serum concentration of MDA progressively increases with severity of atherosclerotic changes in the carotid arteries within both groups of patients. On the other hand, we have also shown significant changes in serum concentration of MDA between the different subgroups of CKD and PD patients according to the status of atherosclerotic changes in the carotid arteries. The highest levels of serum MDA were in stage AS3 in both groups of patients. Significant correlation was confirmed between serum concentration of MDA with IMT-CA and plaque score in all study patients. In addition, our results indicate that the level of serum MDA through all the stages of atherosclerosis was significantly lower in PD patients in comparison with observed CKD patients treated conservatively, which can be attributed to the influence of preserved residual renal function (RRF) and the absence of trusted signs of inflammation. Since peritoneal dialysis is intrinsic type of dialysis through the peritoneum as biocompatible membrane, this result also suggests the possible influence of peritoneal dialysis on oxidative stress.

A few studies reported increased OS among PD patients, but other prooxidants (TBARS, TAC) were measured, associated with hypoalbuminemic state or loss of residual renal function (RRF), and without comparing it with nondialysis CKD patients [25, 27, 30]. Khaira et al. [31] found that PD patients have markedly impaired endothelial function as documented by impaired flow mediated dilatation of brachial artery with higher serum concentration of OS markers. Furthermore, Raju et al. [29] confirmed significant increase in serum MDA in hemodialysis patients, compared with the same patients before they had started the hemodialysis treatment, which could be attributed to a bioincompatibility of dialysis membrane and diffusion of hydrophilic compounds to the dialysate and influx of endotoxin from the dialysate. These factors lead to activation of macrophages and production of reactive oxygen species (ROS) and also a loss of antioxidants during hemodialysis sessions [26]. All the above factors cause an increase in production of free radicals, peroxidation of lipids, and further rise in serum MDA level after episodes of dialysis [32]. Our results suggest that oxidative stress is one of the factors that mediate the relationship between CKD, atherosclerosis, and CMS.

Activity of MMP-9 is highly associated with the progression of CKD, diabetes, and coronary arterial disease [33, 34]. This MMP is secreted by inflammatory cells in the adventitia or smooth muscle cells in the media. The degraded elastic fiber induces calcium deposition, which in conjunction with altered vascular structure is associated with vessel stiffening [35, 36]. In CKD arterial stiffening increases cardiac afterload, left ventricular hypertrophy, reduces coronary artery perfusion and myocardial ischaemia, and increases pulse pressure that promotes atheroma formation and vascular remodeling [37].

In our study, no significant difference was found in the serum concentration of MMP-9 between CKD patients treated conservatively and PD patients, but the significant increase was found in the value of this biomarker within both groups with progression of atherosclerotic process. These findings are in line with the findings of Addabbo and associates, who have demonstrated that MMP-9 levels strongly correlated with carotid atherosclerosis burden irrespective of other factors in early, moderate, and advanced CKD [38].

Our data also showed the decreased serum concentrations of MMP-9 in the stage AS1/2 of atherosclerosis in PD patients compared with CKD patients without dialysis treatment. Possible reason of such decrease is unknown. However, it is our belief that the decrease in MMP-9 concentration in AS1/2 stage of PD patients might occur due to the impact of peritoneal dialysis on MMP-9 serum concentration. MMP-9 was significantly higher in stage AS3 in PD patients compared with CKD patients treated conservatively. To our knowledge, the impact of peritoneal dialysis on the concentration MMP-9 has not yet been clarified. Some authors have demonstrated that the hemodialysis process leads to a reduction in plasma concentration of MMP-9 in some patients [38].

A significant correlation of MMP-9 serum concentration with CA-IMT and plaque score was found in all monitored patients in this study. Also, in multiple regression model we confirmed a significant independent association of MDA and MMP-9 with these ultrasonography parameters characterizing arterial wall and atherosclerotic changes of carotid arteries.

The need for identification of risk factors and serum markers of atherosclerosis in the process of early detection and prediction of risk for cardiovascular disease has attracted a lot of attention in recent years. The significantly lower levels of MDA in PD-CMS patients in comparison with CKD-CMS patients not yet dialysis-dependent and its increase with atherosclerosis progression, as well as obtained higher values of MMP-9 during progression of atherosclerosis especially in PD patients, could be a new contributing factor of our study.

However, this study has certain limitations, such as the small number of respondents and a cross-sectional design of study. A question on the periodontal disease status of those patients was not included, although periodontal disease and kidney disease are highly associated and periodontal disease is alone associated with increases in MMP-9 [39]. Whether the longitudinal profile of MDA and MMP-9 in CKD nondialysis-dependent patients and PD patients, both with signs of CMS, provides additional information on the predictive power of these biomarkers for the progression of atherosclerosis remains a question for testing in future longitudinal studies with a larger number of patients.

## 5. Conclusion

The results suggest that MDA and MMP-9 could be mediators of CKD-related vascular remodeling in CMS. The study data also suggest that that factors mediating relationship between cardiometabolic syndrome, chronic kidney disease, and atherosclerosis can include malondialdehyde and MMP-9.

## Figures and Tables

**Table 1:** Basal characteristics of monitored patients.

	Control subjects (20)	CKD and PD pts (52)	*P*	CKD pts with eGFR < 60 > 15 mL/min/1.73 m^2^ (30)	PD pts (22)	*P*
Age (years)	57.8 ± 14.2	59.8 ± 161	>0.05	63.6 ± 15.1	54.7 ± 16.2	>0.05
Smokers (yes)	35%	46%	>0.05	52%	38%	>0.05
SBP (mmHg)	124 ± 8.68	140.5 ± 19.65	<0.01	135.3 ± 20.44	147.6 ± 16.4	<0.05
DBP (mmHg)	79.8 ± 5.5	90.5 ± 11.22	<0.05	83.7 ± 10.67	90.7 ± 11.21	<0.05
Hemoglobin (g/L)	146.3 ± 11.82	117.3 ± 22.48	<0.01	126.4 ± 23.92	104.7 ± 12.2	<0.01
Albumins (g/L)	37.6 ± 1.76	34.4 ± 6.02	>0.05	36.8 ± 5.83	31.1 ± 4.59	<0.01
CRP (mg/L)	2.8 ± 1.96	9.2 ± 8.81	<0.01	9.8 ± 9.15	8.4 ± 8.48	>0.05
Cholesterol (mmol/L)	5.2 ± 1.37	5.7 ± 0.81	>0.05	4.9 ± 1.35	5.8 ± 1.26	>0.05
Triglycerides (mmol/L)	1.9 ± 0.15	2.0 ± 1.19	>0.05	1.8 ± 0.79	2.4 ± 1.52	>0.05
Uric acid (*µ*mol/L)	272.9 ± 53.1	362.7 ± 104.2	<0.01	364.7 ± 120.8	360 ± 79.9	<0.01
BMI (kg/m^2^)	22.3 ± 2.2	26.6 ± 2.95	>0.05	26.9 ± 3.7	25.7 ± 2.2	>0.05
MDA (ng/mL)	24.9 (12.3–26.9)	37 (24.1–47.7)	<0.01	37 (27–47.1)	35.9 (23.7–47.7)	>0.05
MMP-9 (ng/mL)	321.9 (24–370.9)	419.4 (350.9–447)	<0.01	420 (390.1–442)	390 (350.9–464)	>0.05

Results are expressed as mean ± standard deviation or median and interquartile range (25%–75%); pts: patients; CKD: chronic kidney disease; PD: peritoneal dialysis; SBP: systolic blood pressure; DBP: diastolic blood pressure; CRP: C-reactive protein; BMI: body mass index.

**Table 2:** The serum concentration of MDA and MMP-9 in different patient subgroups according to atherosclerotic stage.

	AS0	AS1/2	AS3	*P*
CKD				
MDA (ng/mL)	24.1 (18.8–27)	40.3 (31.9–45.6)	74.3 (68.2–76.3)	<0.01
MMP-9 (ng/mL)	320 (250–350)	425.2 (405.5–439.5)	462.4 (458.1–464.2)	<0.01

PD				
MDA (ng/mL)	20.6 (10.4–31.3)	35.6 (28.3–39.1)	53.9 (49.8–63.2)	<0.01
MMP-9 (ng/mL)	369.4 (350.5–432.6)	374.8 (346.4–397.7)	469.3 (464–480.6)	<0.01

**Table 3:** Multiple regression model of study biomarkers and their relation with indicators of carotid atherosclerosis in observed CKD and PD patients.

	*B*	SE	Beta	*t*	*P* value	CI 95% lower–higher
MDA (ng/mL)	0.005	0.001	0.675	6.595	**0.000**	0.004–0.007
MMP-9 (ng/mL)	0.001	0.000	0.254	2.480	**0.017**	0.000–0.001
Dependent variable: IMT						

MDA (ng/mL)	0.029	0.004	0.683	6.941	**0.000**	0.020–0.037
MMP-9 (ng/mL)	0.003	0.001	0.255	2.592	**0.013**	0.001–0.005
Dependent variable: plaque score						

SE: standard error; CI: confidence interval: IMT: intima media thickness; MDA: malondialdehyde; MMP-9: matrix metalloproteinase-9.

## Abbreviations

MDA: Malondialdehyde
MMP-9: Matrix metalloproteinase-9
eGFR: Estimated glomerular filtration rate
CKD: Chronic kidney disease
PD: Peritoneal dialysis
CVD: Cardiovascular disease
CMS: Cardiometabolic syndrome
TBARS: Thiobarbituric acid reactive substances
TAC: Total antioxidant capacity
ROS: Reactive oxygen species
AGE: Advanced glycosylated end products
OS: Oxidative stress
ECM: Extracellular matrix
IMT: Intima media thickness
PS: Plaque score
pt: Patient
CA: Carotid artery
AS: Atherosclerosis
PSV: Peak systolic velocity.
